# Volumentherapie – welches Präparat in welcher Situation?

**DOI:** 10.1007/s00063-024-01194-0

**Published:** 2024-10-09

**Authors:** Timo Mayerhöfer, Georg F. Lehner, Michael Joannidis

**Affiliations:** Gemeinsame Einrichtung für Intensiv- und Notfallmedizin, Department für Innere Medizin, Medizinische Universität Innsbruck, Anichstraße 35, 6020 Innsbruck, Österreich

**Keywords:** Balancierte Kristalloide, Kochsalzlösung, Kolloide, Humanalbumin, Sepsis, Crystalloid solutions, balanced, Normal saline solution, Colloids, Serum albumin, human, Sepsis

## Abstract

Die gängigen zur Volumentherapie verwendeten Lösungen sind Kristalloide und Kolloide. Kristalloide lassen sich in 0,9 % Natriumchlorid (NaCl) und balancierte Kristalloide (BK) unterteilen. Kolloide können in künstliche/artifizielle Kolloide und Humanalbumin (als natürliches Kolloid) unterteilt werden. Große Studien konnten Vorteile von BK gegenüber 0,9 % NaCl in Bezug auf renale Endpunkte zeigen, was vor allem durch den unphysiologisch hohen Chloridgehalt in 0,9 % NaCl bedingt sein dürfte. Weitere Studien wie BaSICS und PLUS Trial zeigten an einem heterogenen Kollektiv keine signifikanten Unterschiede in der Mortalität. Jedoch deuten Metaanalysen auf Vorteile der BK hin. Insbesondere bei Patient:innen mit erhöhtem Risiko für eine akute Nierenschädigung, Azidose und/oder Hyperchlorämie sollten daher primär BK verwendet werden. Außer für spezielle Indikationen, so etwa bei Patient:innen mit Leberzirrhose oder „resuscitation“ in der Sepsis nach initialer Volumentherapie mit BK, sollte Albumin nicht eingesetzt werden. Andere, artifizielle, Kolloide sollten mit Zurückhaltung verabreicht werden. Für Hydroxyethylstärke gibt es eindeutige Schädigungssignale bei Intensivpatient:innen.

## Abstract (en)

The most commonly used fluids for volume therapy are crystalloids and colloids. Crystalloids comprise 0.9% sodium chloride and balanced crystalloids (BC). Colloids can be divided into artificial colloids and human albumin (a natural colloid). Large studies show advantages for BC over 0.9% NaCl with respect to renal endpoints, probably due to the unphysiologically high chloride content of 0.9% NaCl. However, other studies, such as the BaSICS and PLUS trials, showed no significant differences in mortality in a heterogeneous population. Despite this, meta-analyses suggest advantages for BC. Therefore, BC should be preferred, especially in patients at increased risk of acute kidney injury, with acidemia and/or hyperchloremia. Except for specific indications (e.g., in patients with cirrhosis, sepsis resuscitation after initial volume therapy with BC), albumin should not be used. There is clear evidence of harm from hydroxyethyl starch in intensive care patients.

Die Verabreichung intravenöser Flüssigkeit ist eine der häufigsten Interventionen bei kritisch kranken Patient:innen. Generell kann dabei vor allem in der Resuscitation-Phase zwischen Kolloiden und Kristalloiden unterschieden werden, wobei Letztere noch einmal in zwei große Gruppen unterteilt werden können: zum einen 0,9 %ige Natriumchloridlösung (NaCl, auch als isotone Kochsalzlösung oder im Englischen als „normal saline“ oder „isotonic saline“ bezeichnet), zum anderen balancierte Kristalloide (BK). Bei den Kolloiden kann im Wesentlichen zwischen artifiziellen Kolloiden und Humanalbumin (als Vertreter natürlicher Kolloide) unterschieden werden. Des Weiteren sind 5 %ige Glukose und halbisotone Lösungen zu nennen, die jedoch aufgrund eines deutlich geringeren Volumeneffekts vor allem in der sogenannten „maintenance“ (Erhaltung) von Bedeutung sind und als Resuscitation-Flüssigkeit keine Rolle spielen.

Im Folgenden werden wichtige pathophysiologische Überlegungen erörtert und große klinische Studien zu den möglichen Effekten des jeweiligen Volumens bei kritisch Kranken zusammengefasst.

## Pathophysiologische Grundlagen

### Köperwasser und Volumenverteilung

**Abb. 1:**
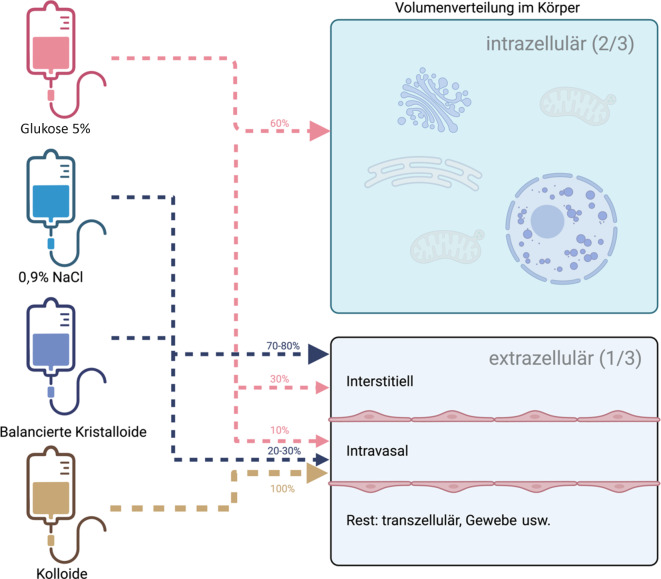
Theoretische (ungefähre) Verteilung intravenöser Flüssigkeiten bei gesunden Menschen. (Modifiziert nach [2]; erstellt mit BioRender.com)

Das gesamte Wasser im Körper eines Menschen befindet sich zu etwa zwei Dritteln intrazellulär und zu etwa einem Drittel im sogenannten Extrazellulärraum, der sich wiederum in den intravasalen Raum, den interstitiellen Raum, den transzellulären Raum und die Lymphe unterteilen lässt [1]. Kristalloide verteilen sich zunächst im Extrazellulärraum auf den intravasalen Raum und den interstitiellen Raum (Abb. 1). Aufgrund ihrer annähernd isoosmotischen Eigenschaft und der Tatsache, dass Natrium nicht die Zellwand passieren kann, gelangen sie nicht nach intrazellulär – im Gegensatz zu 5 %igen Glukoselösungen, deren Glukose von Zellen aufgenommen wird. Dadurch bleibt freies Wasser zurück und verteilt sich auf den intrazellulären, intravasalen und interstitiellen Raum (Abb. 1). Der intravasale Raum (Plasma) und der interstitielle Raum sind durch Kapillaren voneinander getrennt [2].

Wie schnell die Verteilung von intravasal nach interstitiell abläuft, hängt von vielen Faktoren ab. Lange ist man davon ausgegangen, dass hier die Kapillaren als semipermeable Membran die Hauptrolle spielen und so Flüssigkeit von interstitiell nach intravasal absorbiert bzw. ausgetauscht wird [3, 4]. Mittlerweile ist klar, dass vor allem die endotheliale Glykokalyx eine entscheidende Rolle spielt, die ein dynamisches Konstrukt darstellt, das beispielsweise besonders bei der Sepsis und beim septischen Schock abgebaut und dysfunktional wird [5]. In einer Studie von Hippensteel et al. [6] konnte allerdings auch gezeigt werden, dass dieser Abbau durch eine intravenöse Flüssigkeitstherapie sogar verstärkt wird und proportional zum verabreichten Volumen ist.

### Säure-Basen-Haushalt und tubuloglomeruläres Feedback

Ziel der initialen Flüssigkeitstherapie („resuscitation“) ist meist, Volumenverluste auszugleichen und für eine adäquate Organperfusion zu sorgen. Die gängigste dafür zur Verfügung stehende Flüssigkeit ist isotones 0,9 % NaCl; es enthält als Gegenspieler zu den 154 mmol/l Natrium (Kation) 154 mmol/l Chlorid (Anion). BK sollen die Eigenschaften des Blutplasmas nachahmen und enthalten statt des unphysiologisch hohen Chloridanteils in 0,9 % NaCl zusätzlich einen anderen Puffer, beispielsweise Laktat oder Acetat, und andere Elektrolyte, wie Kalium, Kalzium oder Magnesium (Tab. 1; [7]). Bedenken bezüglich des Einsatzes von 0,9 % NaCl gibt es schon sehr lange [7]; sie hängen vor allem mit dem Einfluss des Chloridgehalts auf den Säure-Basen-Haushalt zusammen. Der hohe Chloridgehalt kann zu einer hyperchlorämischen metabolischen Azidose führen, wie in Tierexperimenten gezeigt wurde [8]. Die Regulierung des pH-Werts im Organismus erfolgt in einem komplexen Zusammenspiel von Puffersystemen, Niere und Lunge. Gemäß dem Stewart-Modell wird der pH-Wert im Blut vor allem vom pCO_2_, der Konzentration von schwachen Säuren (beispielsweise Albumin, Phosphat) und der sogenannten „strong ion difference“ (SID) bestimmt. Die SID berechnet sich aus dem Unterschied der starken Kationen (hauptsächlich Natrium, Kalium, Kalzium und Magnesium) und Anionen (hauptsächlich Chlorid). Bei der Verabreichung von 0,9 % NaCl steigt vor allem der Chloridanteil im Plasma stärker an als das Natrium. Dies führt zur Verringerung der SID und somit zu einer reduzierten positiven Nettoladung. Daraus folgt eine kompensatorische Reaktion mit Protonenbildung, um das Gleichgewicht wiederherzustellen, was zu einer Azidose führt [7, 9].

**Tab. 1:** Zusammensetzung gängiger in Deutschland und Österreich verfügbarer Kristalloide und die Normwerte im menschlichen Plasma

Komponenten (mmol/l)	Ringer-Laktat	Sterofundin ISO	Jonosteril	Ringer-Acetat	Plasma-Lyte	ELO-MEL	0,9 % NaCl	Normwerte menschliches Plasma
Natrium	130,0	140	137	130,0	140,0	140,0	154	135–145
Kalium	4,0	5	4	4,0	5,0	5,0	0	3,5–5,0
Kalzium	1,5	2	1,65	1,5	0,0	2,5	0	2,1–2,6
Magnesium	0,0	1	1,25	0,0	1,5	1,5	0	0,7–1,0
Chlorid	109	127	110	109	98	108	154	95–105
Laktat	28,0	0	0	0,0	0,0	0,0	0	0,5–2,2
Acetat	0	24	36,8	24,0	27,0	45	0	0
Glukonat	0	0	0	0	23	0	0	0
Theoretische Osmolarität (mOsm/l)	273	309	0	273	294,0	302	308	275–295

Einige Lösungen sind von mehreren Herstellern verfügbar, weswegen die Angaben geringfügig abweichen können

Der hohe Chloridgehalt von 0,9 % NaCl wirkt sich direkt negativ auf die renale Durchblutung aus

**Abb. 2:**
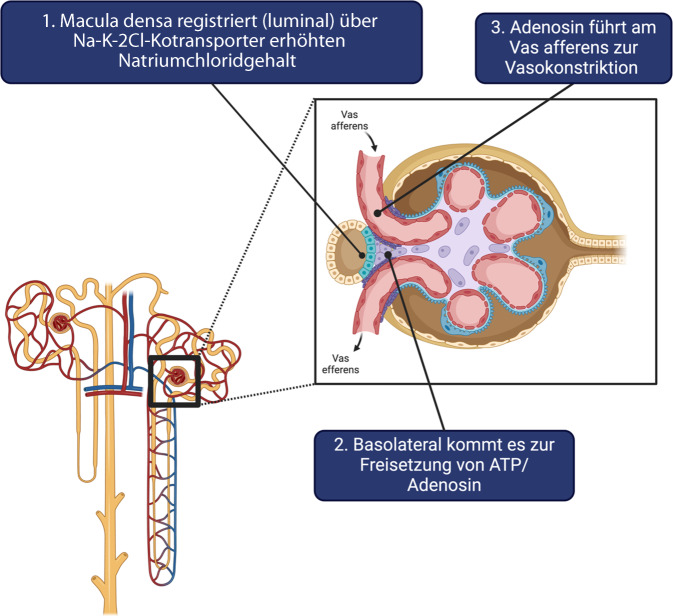
Vereinfachte Darstellung des tubuloglomerulären Feedbackmechanismus. Die Macula densa im aufsteigenden Teil der Henle-Schleife registriert zunächst über den Na-K-2Cl-Kotransporter auf der luminalen Seite den erhöhten Chlorid- bzw. Natriumchloridgehalt (1), was zur Freisetzung von ATP auf der basolateralen Seite führt (2). Dies wiederum führt über einen Adenosinrezeptor in weiterer Folge zur Vasokonstriktion des Vas afferens (3) und somit zur Reduktion des renalen Blutflusses. *ATP* Adenosintriphosphat. (Erstellt mit BioRender.com)

Darüber hinaus hat der hohe Chloridgehalt einen direkten negativen Einfluss auf die renale Durchblutung [10], ein Effekt, der sich pathophysiologisch mit dem tubuloglomerulären Feedback erklären lässt (Abb. 2).

Neben der Veränderung des renalen Blutflusses [10] hat eine durch 0,9 % NaCl ausgelöste hyperchlorämische metabolische Azidose zahlreiche weitere negative Effekte: Tierexperimentelle Untersuchungen konnten eine gastrointestinale Schädigung [11], verstärkte Inflammation [12] sowie Koagulopathie [13] zeigen.

### Osmolarität

Einen weiteren wesentlichen Unterschied, neben der Elektrolytzusammensetzung, stellt die unterschiedliche Osmolarität der verschiedenen Lösungen dar (Tab. 1). Durch den höheren Natriumanteil ist die theoretische Osmolarität bei 0,9 % NaCl höher als bei den BK. Dies spielt vor allem bei Patient:innen mit potenziell erhöhtem intrakraniellem Druck eine entscheidende Rolle, bei dem eine Erniedrigung der Osmolarität unbedingt vermieden werden sollte, um einen weiteren Anstieg des Hirndrucks zu verhindern. Bei einer Flüssigkeitstherapie bietet hier 0,9 % NaCl theoretische Vorteile gegenüber BK. In einer Arbeit an gesunden Freiwilligen konnte bereits 1999 gezeigt werden, dass bei Verabreichung von Ringer-Laktat die Osmolarität im Blut sinkt, nicht jedoch bei Verabreichung von 0,9 % NaCl [14].

## Balancierte Kristalloide und 0,9 % NaCl

Ob die Verwendung von BK oder 0,9 % NaCl einen Effekt auf harte klinische Endpunkte hat, wurde mittlerweile in mehreren großen Studien untersucht. Obwohl in nichtselektierten Populationen keine signifikanten Unterschiede in der Mortalität beobachtet wurden, können die jeweiligen Effekte von BK und 0,9 % NaCl aufgrund von deren breiter Anwendung dennoch von Bedeutung sein; unter Umständen sind sie für bestimmte Patient:innengruppen entscheidend.

### Gesunde Proband:innen

In der bereits erwähnten Arbeit zur Osmolarität bei gesunden Freiwilligen war die Verabreichung von 0,9 % NaCl auch mit dem Auftreten einer Azidose assoziiert. Während die Osmolarität wieder den Ausgangswert annahm, bestand die pH-Veränderung fort [14]. In einer weiteren randomisierten, kontrollierten Studie an 12 gesunden männlichen Probanden, denen jeweils zu unterschiedlichen Zeitpunkten 2 l 0,9 % NaCl und 2 l eines BK verabreicht wurden, ging die Infusion von 0,9 % NaCl im Vergleich zu BK mit einer Reduktion der renalen Blutflussgeschwindigkeit sowie mit einer Reduktion der kortikalen Perfusion einher [15].

### Chirurgisches Setting

Zur Frage, ob eine der beiden Lösungen Vorteile im perioperativen Management bietet, existieren einige randomisierte, kontrollierte Studien in verschiedenen Settings (Sectio [16], Neurochirurgie [17], große abdominelle Eingriffe [18]), die zwar keinen klaren Nachteil von 0,9 % NaCl in Bezug auf das klinische Outcome zeigten, jedoch meist kleine Populationen einschlossen und Säure-Basen-Parameter als primären Endpunkt untersuchten. Die große LICRA-Studie an 1136 herzchirurgischen Patient:innen, die chloridreiche (0,9 % NaCl, 4 % Albumin) gegenüber chloridarmen Flüssigkeiten (BK, 20 % Albumin) untersuchte, konnte keinen signifikanten Unterschied in Bezug auf die Nierenfunktion feststellen, weißt jedoch auch einige Limitationen auf, beispielsweise Unterschiede in den Baseline-Charakteristika [19].

Aufgrund des beschriebenen Mechanismus stellen Patient:innen während bzw. kurz nach einer Nierentransplantation ein besonders gefährdetes Patient:innenkollektiv dar. Randomisierte Studien zeigen, dass die Verabreichung von 0,9 % NaCl auch in dieser Gruppe zu einer metabolischen Azidose führt [20–24]. Außerdem war in einigen Arbeiten eine Hyperkaliämie seltener, wenn BK verabreicht wurden [25, 26]. Im klinischen Alltag bestehen bei Patient:innen mit Hyperkaliämie oft Vorbehalte gegenüber der Verwendung von BK aufgrund ihres (geringen) Kaliumgehalts (Tab. 1). Die Daten deuten darauf hin, dass der Shift durch die 0,9 %-NaCl-bedingte (verstärkte) Azidose jedoch eine größere Rolle spielt und BK hier Vorteile bieten [7]. In einer aktuellen Arbeit von 2023, der BEST-Fluids-Studie, konnte die Verabreichung von BK gegenüber 0,9 % NaCl das Auftreten einer verzögerten Transplantatfunktion reduzieren [27].

### Notaufnahme und Intensivstation

Es existieren einige Beobachtungsstudien mit verschiedenen Populationen [28–30] zum Vergleich von balancierten Kristalloiden und 0,9 % NaCl im Bereich der Notfall- und Intensivmedizin, die eine Assoziation des erhöhten Chloridgehalts von 0,9 % NaCl mit der Mortalität zeigen. Eine erste Interventionsstudie von 2012, in der nach einer initialen Phase mit „Standardflüssigkeitstherapie“ chloridreiche Lösungen auf den teilnehmenden Stationen vermieden wurden, zeigte, dass in der zweiten Phase die Inzidenz einer akuten Nierenschädigung („acute kidney injury“ [AKI]) und die Anwendung einer Nierenersatztherapie signifikant reduziert waren [30]. Auf diese Arbeit folgte zunächst 2015 die SPLIT-Studie, die in 4 neuseeländischen Intensivstationen 2278 Patient:innen einschloss. Es gab keine signifikanten Unterschiede im primären Endpunkt (AKI). Neben der für die Detektion kleiner Unterschiede zu geringen Fallzahl hatte die Studie noch andere Limitationen, wie etwa die geringe Sterblichkeit und den hauptsächlichen Einschluss von Patient:innen mit elektiven Operationen [31].

Es folgten insgesamt 4 deutlich größere randomisierte, kontrollierte Studien (Tab. 2). Die SMART- und die SALT-ED-Studie wurden am selben Zentrum durchgeführt und hatten ein sehr ähnliches Design mit einer nichtverblindeten Clusterrandomisierung, wobei jeweils zunächst die eine Flüssigkeit für einen Monat und anschließend die andere Flüssigkeit über eine gewisse Zeit verabreicht wurde. In der SALT-ED-Studie wurden nicht kritisch kranke Notaufnahmepatient:innen eingeschlossen. Im primären Endpunkt (krankenhausfreie Tage) zeigte sich kein signifikanter Unterschied. Die Rate an „major adverse kidney events“ (sekundärer Endpunkt) war jedoch bei den Patient:innen, die BK erhielten, niedriger [32]. Ähnliches zeigte sich in der SMART-Studie, die nur Patient:innen der Intensivstation einschloss; der primäre Endpunkt („major adverse kidney events“ an Tag 30) war hier in der BK-Gruppe signifikant reduziert [7]. Interessanterweise war dieser Unterschied umso größer, je größer das verabreichte Volumen war.

**Tab. 2:** Übersicht über die größten randomisierten, kontrollierten Studien, die balancierte Kristalloide und 0,9 % NaCl vergleichen. (Modifiziert nach [43])

Studienname	Studiendesign	Population	Endpunkt	Ergebnis
SMART (2018)	Multizentrische (5 ICU, 1 akademisches Zentrum), clusterrandomisierte Multiple-cross-over-Studie	15.802 erwachsene ICU-Patient:innen	MAKE innerhalb von 30 Tagen	Balancierte Kristalloide reduzierten die Häufigkeit von MAKE
SALT-ED (2018)	Single-center‑, Multiple-cross-over-Studie	13.347 erwachsene Notaufnahmepatient:innen (nicht kritisch krank)	Krankenhausfreie Tage bis Tag 28	Kein signifikanter Unterschied in den krankenhausfreien Tagen, niedrigere MAKE-Rate
BaSICS (2021)	Multizentrische, randomisierte, kontrollierte Studie	11.052 erwachsene ICU-Patient:innen	90-Tage-Mortalität	Kein signifikanter Unterschied in der 90-Tage-Mortalität
PLUS (2022)	Multizentrische, randomisierte, kontrollierte Studie	5037 erwachsene ICU-Patient:innen	90-Tage-Mortalität	Kein signifikanter Unterschied in der 90-Tage-Mortalität

*ICU* „intensive care unit“ (Intensivstation), *MAKE* „major adverse kidney events“

Es folgte der BaSICS Trial, der keine Unterschiede zwischen den beiden verwendeten Flüssigkeiten hinsichtlich der 90-Tage-Mortalität zeigen konnte. Auch diese Studie hatte einige Limitationen, wobei vor allem die Verabreichung von nicht im Studiendesign berücksichtigter Flüssigkeit und die insgesamt geringe Menge (unter 1 l/Tag) zu nennen sind [33]. In einer Sekundäranalyse wurde insbesondere die vor der Randomisierung verabreichte Flüssigkeit mituntersucht und es zeigte sich, dass Patient:innen, die nur BK erhalten, sehr wahrscheinlich im Sinne einer besseren 90-Tage-Mortalität profitieren [34]. Allerdings zeigte 0,9 % NaCl auch in dieser Studie ein vorteilhaftes Signal in der Gruppe von Patient:innen mit *Schädel-Hirn-Trauma*. Dieser Effekt scheint durch die höhere Osmolarität von 0,9 % NaCl bedingt zu sein (Tab. 1). Im Jahr 2022 ergab schließlich die PLUS-Studie keine signifikanten Unterschiede bei der 90-Tage-Mortalität [35]. Zeitgleich mit dieser Arbeit wurde eine Metaanalyse publiziert, die 13 Studien mit insgesamt 35.884 Patient:innen einschloss. Das Resümee dieser Analyse ist, dass sich der durchschnittliche Effekt von BK mit hoher Wahrscheinlichkeit positiv auf die Mortalität auswirkt [36].

**Abb. 3:**
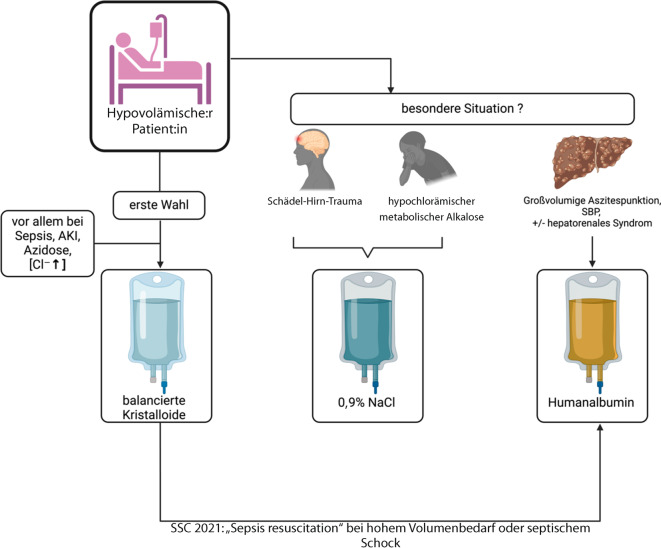
Schematische Darstellung zur Wahl des Präparats für die Volumentherapie bei kritisch kranken hypovolämischen Patient:innen. *AKI* „acute kidney injury“ (akute Nierenschädigung), *NaCl* Natriumchlorid, *SBP* spontan bakterielle Peritonitis, *SSC* Surviving Sepsis Campaign. (Erstellt mit BioRender.com)

Sowohl deutsche [37] als auch europäische Leitlinien [38] kommen daher zu dem Schluss, dass BK bei kritisch Kranken zu bevorzugen sind. Ausnahmen stellen spezifische Gruppen von Patient:innen dar, beispielsweise mit Schädel-Hirn-Trauma oder hypochlorämischer metabolischer Alkalose (meist nach massivem Erbrechen), bei denen primär 0,9 % NaCl eingesetzt werden sollte. Auch in der *Sepsis*, einer häufigen Entität, bei der die Flüssigkeitstherapie eine wichtige Rolle spielt, empfehlen die aktuellen Surviving-Sepsis-Campaign(SSC)-Leitlinien als primäre Resuscitation-Flüssigkeit BK (Abb. 3; [39]). Diese schwache Empfehlung basierte im Wesentlichen auf einer Sekundäranalyse der SMART-Studie, in der BK im Vergleich zu 0,9 % NaCl in der Subgruppe der Patient:innen mit Sepsis zu einer niedrigeren Mortalität und weniger AKI führten [40]. Auch eine Sekundäranalyse des BaSICS Trial legt nahe, dass, insbesondere wenn viel Flüssigkeit verabreicht wird, diese bei Patient:innen mit Sepsis Einfluss auf die 90-Tage-Mortalität hat [41].

Leitlinien empfehlen balancierte Kristalloide bei kritisch Kranken – mit Ausnahmen – zu bevorzugen

Kurz soll hier noch auf die Maintenance-Phase eingegangen werden, in der oftmals auch Kristalloide zum Einsatz kommen. Ziel dieser Phase ist es, nach initialer Stabilisierung das Extrazellulärvolumen zu erhalten und die Elektrolyte im Normalbereich zu halten [42]. In den meisten Fällen ist der Großteil des Bedarfs einerseits durch Flüssigkeiten, die bei der Medikamentenverabreichung benötigt werden, und andererseits durch die Ernährung gedeckt; eine zusätzliche spezielle parenterale Flüssigkeitstherapie ist nur selten nötig [13]. Eine Flüssigkeitsgabe mit der Indikation „maintenance“ sollte nur dann durchgeführt werden, wenn Patient:innen enteral nicht genug Flüssigkeit aufnehmen können. Die meisten der verfügbaren Empfehlungen beruhen auf Expertenmeinungen. Da eine ständige Verabreichung von Kristalloiden mittel- bis langfristig das Risiko einer Hypernatriämie und Hyperchlorämie birgt, kommen hier teilweise halbisotone Lösungen zum Einsatz, die wiederum mit dem Risiko einer Hyponatriämie einhergehen [42].

## Artifizielle und natürliche Kolloide

Kolloidale Lösungen wie Hydroxyethylstärke (HES) und Albumin haben eine höhere osmotische Aktivität und bieten daher den theoretischen Vorteil einer effektiveren Volumenexpansion (Abb. 1).

### Hydroxyethylstärke

Es gibt einige groß angelegte Studien, von denen insbesondere CHEST (6 % HES vs. 0,9 % NaCl), der 6S Trial (HES 130/0,42 vs. Ringer-Acetat) und VISEP hervorzuheben sind, die ein Schadenssignal, meist im Sinne einer renalen Schädigung, zeigten [43]. Während eine Cochrane-Metaanalyse insgesamt keinen Vor- oder Nachteil gegenüber anderen Kolloiden ergab [44], wurde in anderen Arbeiten geschlussfolgert, dass HES in allen Situationen das Risiko für AKI und Nierenersatztherapie erhöht, weswegen der Einsatz vermieden werden sollte [45]. Die European Medicines Agency (EMA) und die US Food and Drug Administration (FDA) haben daher jeweils Warnungen veröffentlicht [46, 47], weshalb in Abwägung von Nutzen und Risiko keine HES verwendet werden sollte.

### Gelatine

Gelatine besteht aus Polypeptiden, die aus Rinderkollagen gewonnen werden. Für Gelatine existieren trotz breitem Einsatz keine groß angelegten randomisierten, kontrollierten Studien. Während eine Metaanalyse ein erhöhtes Anaphylaxierisiko und einen Trend zur höheren Mortalität zeigte [48], konnte dies in anderen Arbeiten nicht bestätigt werden [49, 50]. Die meisten Metaanalysen kommen allerdings zu dem Schluss, dass die Evidenz derzeit nicht ausreicht, um eine Aussage über Sicherheit und Effektivität von Gelatine zu treffen. Eine gerade laufende randomisierte, doppelblinde Studie zu Gelatine versucht daher, diese Fragen zu beantworten [51].

### Humanalbumin

Humanalbumin bietet als natürliches Kolloid theoretische Vorteile und insgesamt ein wesentlich geringeres potenzielles Anaphylaxierisiko. Albumin wird in der Leber produziert und hat neben der Erhaltung des osmotischen Drucks noch weitere Aufgaben, beispielsweise im Säure-Basen-Haushalt oder als Transportprotein [52]. Ihm werden auch antiinflammatorische Eigenschaften nachgesagt. Klare Empfehlungen mit entsprechender Datengrundlage gibt es vor allem bei Patient:innen mit Zirrhose, etwa nach großvolumiger Aszitespunktion, und bei der spontan bakteriellen Peritonitis [53].

Bei Sepsis und hohem Volumenbedarf wird die Gabe von Albumin zusätzlich zu Kristalloiden empfohlen

Die größte randomisierte Studie zu Humanalbumin beim kritisch Kranken ist die SAFE-Studie, in die 6997 kritisch kranke Patient:innen eingeschlossen wurden. Hierbei wurde 4 % hypoonkotisches Humanalbumin mit 0,9 % NaCl verglichen. In der Humanalbumingruppe wurde zwar insgesamt weniger Flüssigkeit benötigt (etwa im Verhältnis 1:1,4), jedoch zeigten sich keine Unterschiede in der Mortalität [54]. Eine Post-hoc-Analyse ergab eine erhöhte Mortalität in der Humanalbumingruppe im Vergleich zur 0,9 %-NaCl-Gruppe für Patientinnen mit Schädel-Hirn-Trauma [55]. Wie auch im BaSICS Trial lässt sich dies sehr wahrscheinlich mit der höheren Osmolarität von NaCl im Vergleich zu 4 % hypoonkotischem Humanalbumin erklären [56–58].

Aufgrund eines positiven Signals bei Patient:innen mit schwerer Sepsis in der SAFE-Studie folgte die ALBIOS-Studie, in der ausschließlich Patient:innen mit Sepsis eingeschlossen wurden. Auch hier zeigte sich kein signifikanter Unterschied in Bezug auf den primären Endpunkt, jedoch ein positives Signal für Patient:innen im septischen Schock [59]. Unter Einbezug einer weiteren Arbeit (EARSS), deren Ergebnisse seit Langem leider nur als Abstract vorliegen, konnte in einer gepoolten Analyse ein Vorteil für Patient:innen mit schwerer Sepsis gezeigt werden [60]. Aktuell wird in den SSC-Leitlinien von 2021 mit moderater Evidenzqualität empfohlen, bei Sepsis und hohem Volumenbedarf zusätzlich zu Kristalloiden Albumin zu verwenden (Abb. 3). Es gibt zwar keinen etablierten Cut-off für den Beginn der Albumingabe, aber der Einsatz scheint bei Vorliegen eines septischen Schocks und erfolgter Volumentherapie mit BK gerechtfertigt zu sein, zumal beim septischen Schock sowohl in der Post-hoc-Subgruppenanalyse der ALBIOS-Studie [59] als auch in einer Metaanalyse ein Nutzen hinsichtlich der Mortalität gezeigt wurde [61]. Aktuell läuft außerdem die ARISS-Studie, die Patient:innen mit septischem Schock einschließt und mehr Klarheit schaffen dürfte ([62]; Tab. 3).

**Tab. 3:** Klinische Beispiele mit Empfehlungen zur Wahl der Flüssigkeit im Falle einer Hypovolämie

Klinisches Szenario	Balancierte Kristalloide	0,9 % NaCl	5 % Glukose	Artifizielle Kolloide (Gelatine)	Humanalbumin
Sepsis/septischer Schock	++	+	/	/	+ (bei hohem Volumenbedarf/im septischen Schock)
Hypovolämischer Schock durch Blutung	++ (nach Verabreichung von Blutprodukten oder wenn diese nicht sofort verfügbar sind)	+	/	(+) /	/
Hypovolämischer Schock durch Dehydratation	++	+	/	/	/
Hypovolämie und Azidose/Hyperchlorämie	++	–	/	/	/
Schädel-Hirn-Trauma/erhöhter intrakranieller Druck	–	++ (ggf. hypertones NaCl)	–	/	– (kein hypoonkotisches Humanalbumin)
Hypernatriämie	/	–	+ oder halbisotones NaCl	/	–
Hypovolämische Hyponatriämie	+	+	–	/	/
AKI	++	– (+ bei Erbrechen und hypochlorämischer Alkalose)	/	/	/
Leberzirrhose + großvolumige Aszitespunktion und/oder SBP	+	+	/	/	++

*++* Empfehlung zur Gabe (meist durch ausreichend Daten belegt), *+* eine Gabe kann erwogen werden, ***–*** eine Gabe ist eher nicht empfohlen, */ *keine typische Indikation bzw. keine ausreichende Datengrundlage

*AKI* „acute kidney injury“ (akute Nierenschädigung), *NaCl* Natriumchlorid, *SBP* spontan bakterielle Peritonitis

## Fazit für die Praxis

- Bei den meisten hypovolämischen Patient:innen stellen balancierte Kristalloide die erste Wahl dar.
- Wichtige Ausnahmen sind Patient:innen mit Schädel-Hirn-Trauma (erhöhter intrakranieller Druck) oder mit metabolischer hypochlorämischer Alkalose (meist nach massivem Erbrechen), bei denen 0,9 % NaCl verwendet werden sollte.
- Albumin sollte nur bei speziellen Indikationen eingesetzt werden, so etwa bei Patient:innen mit Leberzirrhose oder in der Sepsis.
- Hydroxyethylstärke sollte nicht mehr verwendet werden, da einige Studien ein nephrotoxisches Potenzial zeigen. Alle anderen artifiziellen Kolloide sollten mit Zurückhaltung eingesetzt werden, bis große randomisierte Studien ihre Sicherheit und Effektivität belegen.
